# Awake brain surgery: toward optimal cognitive explorations

**DOI:** 10.3389/fnhum.2024.1369462

**Published:** 2024-03-27

**Authors:** Elena Salillas, Serena De Pellegrin, Carlo Semenza

**Affiliations:** ^1^Department of Psychology and Sociology, Universidad de Zaragoza, Zaragoza, Spain; ^2^Neurology Clinic, Department of Neuroscience, Padua University Hospital, Padova, Italy; ^3^Padova Neuroscience Center, University of Padova, Padova, Italy

**Keywords:** awake neurosurgery, direct cortical electrostimulation, cognitive neurosurgery, brain mapping, item sensitivity

## Introduction

*Cognitive Neurosurgery* echoes the knowledge exchange between neurosurgical teams and cognitive sciences (Ojemann, 2003; Landerl et al., 2009; Lang, 2017; Wang et al., 2021). Efforts entail neuroimaging paradigms for presurgical mapping (e.g., Silva et al., 2018; Di Giovanni and Collins, 2023; Papanicolaou, 2023), new protocols for awake surgery (e.g., Rossi et al., 2019; Ius et al., 2021; Gomez-Andres et al., 2022), the study of neuroplasticity after surgery (e.g., Cargnelutti et al., 2020), or the study of cognitive prognosis after surgery (e.g., Mandonnet et al., 2017; Mrah et al., 2022), among others. One of the most critical issues regards the contribution of cognitive neuroscience to the awake surgery process. During awake brain surgery, electrostimulation is applied over the cortex or white matter while the patient performs a cognitive task, provoking a transient disruption in the patient's performance. In this way, functional areas are delimited and preserved. Direct electrostimulation can inform cognitive science as much as the actual knowledge in cognition can inform neurosurgery.

Different journals have devoted special issues to these topics, including ours (La Corte et al., 2022; Montemurro and Trevisi, 2022; Salillas et al., 2022); the potential contributions that neuroscientists can make to improving awake surgery are vast. Here, we want to stress a highly pragmatic aspect: the relevance of focusing on sensitivity in cognitive paradigms.

## One task with non-equivalent items for different brain sites

Some recent reviews have tried to compile the protocols used for surgery (Ruis, 2018; Bu et al., 2021). They involve mainly language paradigms, but not only. And teams vary in the type of tests used. These reviews report commonalities and start a way to generate a specific brain-to-task map that could guide the cognitive part of electrostimulation.

What calls attention is that, in the end, a minimal set of tasks is matched to very different areas in the brain (Bu et al., 2021). For example, picture naming is suggested to be applied to the frontal lobe, the anterior temporal lobe, the posterior temporal lobe, the occipital lobe, and the parietal lobe. That is, virtually to the whole left hemisphere cortex. Furthermore, when addressing the tasks to be applied to white matter stimulation, picture naming is meant to be used to any of the major white matter tracts: the frontal aslant tract, the superior longitudinal fasciculus, the inferior fronto-occipital fasciculus, de uncinate fasciculus, or the inferior longitudinal fasciculus. That is, almost every central white matter tract involved in language. Similarly, semantic association tasks are applied when addressing the frontal lobe, the anterior temporal lobe, or the posterior temporal lobe. Some other tasks appear more specific, such as reading when handling the posterior temporal or occipital lobes or calculation and visuospatial tasks for addressing the parietal lobes.

While stimulation with those tasks to all those sites provokes errors and ultimately delimits functional limits, it is known that those errors will be due to transient damage to very different processes or representations. It is known that the same task (i.e., naming) can elicit various errors, for example, semantic or phonological paraphasias, depending on the stimulated area or tract. This warrants the validity and sensitivity of this production task, which taps different processes. There is even further complexity in verb production items (Rofes and Miceli, 2014). The transiently altered component ultimately defines the commission error.

## Increasing the sensibility of the exploration

The issue we want to put forward is whether we can optimally target specific processes with a proper selection of items within those tasks. There is no need for cognitive experimentation in a delicate surgical context. It should be enough to adapt the cognitive paradigms within a given hospital protocol. It does not disregard implementing new robust known paradigms. Our view here entails a detailed analysis of the items and the errors they provoke. It involves an analysis of the linguistic characteristics of the stimulus and the linguistic characteristics of the errors. Such analysis would likely reveal a systematic association between item profiles and error quality. And what is crucial here is the neural basis of those associations.

On the other hand, the association might also occur between a given stimulated brain area and only a particular type of item (for example, a transitive verb), frequently provoking the same kind of error. This more straightforward pattern can also result in a higher probability of error given an item type and a specific locus. In turn, it is crucial to revise the usual paradigms. The applied standardization only sometimes attends to the whole quality of the items, such as the semantic structure, morphological characteristics, or the argument mapping of verbs. In this way, the brain-to-task map could be refined with specific items within a particular task.

Other tasks like visuospatial processing are less prone to variation across items. Hence, line bisection and some proposed verbal task variations (Bouyer et al., 2023) are sensitive to the single item. As shown below, mathematical cognition is another domain for which items must be scrutinized.

We have exemplified this line of action in three tasks: picture naming, verb generation, and simple calculation.

Gobbo et al. (2021) showed that the semantic characteristics of the nouns used in the picture naming task DO80 (Metz-Lutz et al., 1991) differ in at least one way. The DO80 is based on previously standardized items on name agreement, image agreement, frequency, familiarity, visual complexity, age of acquisition, and level of education (Snodgrass and Vanderwart, 1980; Metz-Lutz et al., 1991). In this case, we focused on the semantic hierarchization of the items and the degree of systematicity in the emission of specific errors. Both items and errors were analyzed using the WordNet database (Roventini et al., 2000). The role of hierarchization in semantics is known (Warrington, 1975; McCarthy and Warrington, 2016), and this is an aspect not considered in tests such as the Boston Naming Test or the D80. Consequently, they are applied in surgery as if the items were equivalent.

In Gobbo et al. (2021), we showed how co-hyponym errors (“lemon” for “orange”) were more likely to be found upon stimulation to temporal areas. Moreover, the number of hyperonyms (i.e., “vehicle” for “car”) predicted the emission of a synonym (“automobile” for “car”) upon inferior frontal lobe stimulation. Hence, it is one of the aspects in which we showed that DO80 items differ, though there are likely more. Other potential differences, such as the phonological complexity of the word in the language at the test, have yet to be assessed. In this case, the dorsal pathway could be a potentially sensitive area.

As per verb generation, another frequently used task, we have recently shown (Salillas et al., 2023) that the restrictions imposed by the eliciting noun can determine the sensitivity to stimulation on the inferior frontal lobe. Following the robust paradigm by Thompson-Schill et al. (1997, 1998) we retrospectively classified the items commonly used in a hospital. The classification attended to the verb selection effort implied by the nouns. Items such as “wheel” require higher selection efforts to finally emit a verb than nouns such as “scissors,” which prompts the verb “cut” after shallow selection efforts. Considering this variable, BA44 and BA45 could be functionally localized, even in right lateralization or reorganization cases. It is an example of how selecting robust paradigms from neurocognitive literature can help gain specificity and locality before surgery. A close analysis of errors committed during inferior frontal stimulation could reveal that most errors are committed after nouns requiring high selection efforts.

Switching to another cognitive domain, mathematical cognition is also frequently assessed when targeting the parietal lobe (Chan-Seng et al., 2014). Concretely, simple calculation is a task often used (Duffau et al., 2002; Roux et al., 2009; Pu et al., 2011; Yu et al., 2011; Della Puppa et al., 2013; Semenza et al., 2017). Here, lateralization might be critical (Semenza et al., 2017; Salillas et al., 2021): we have shown that both hemispheres orchestrate to solve simple calculation processes. While the left hemisphere prefers retrieval mechanisms, the right hemisphere performs approximation during calculation. These conclusions also apply to item scrutiny: when the left parietal areas are stimulated, verifying items with a competing wrong answer (i.e., 2 × 3 = 8) may show more sensitivity and a higher probability of error after stimulation in the left hemisphere. In contrast, verifying items with unrelated answers (i.e., 2 × 3 = 7) may offer more sensitivity when stimulating the right parietal areas.

The three examples we discussed emphasize the opportunity to analyze the errors considering each item and the brain site. Most studies with direct electrostimulation report failure or hit on each item, irrespective of the nature of the errors. However, in some domains, commission errors (i.e., when participants provide a wrong answer instead of a hit or such answers as “I don't know”) are essential. As our examples demonstrate, the analysis of the quality of commission errors provides, in fact, further precious information that can reveal the precise nature of the processes involved. Systematic reports of commission errors, if not throughout analyses, may thus become routine rather than occasional in the future.

Another promising way to explore task/stimuli specifics, in this case before surgery, is using navigated transcranial magnetic stimulation (nTMS). Some studies have done so in object naming, yet a distinction between items has yet to be made. Qualitative analysis of errors has been performed after stimulating the cortex or the white matter tracts (anomia, semantic paraphasia, phonological paraphasia (e.g., Hernandez-Pavon et al., 2014; Vasileiadi et al., 2023), however; no attention has been paid to the actual name requested behind the errors. A retrospective analysis in that line for any TMS study done so far could be a simple and informative advance.

Meta-analytic reviews on TMS studies are also valuable in this respect. A recent meta-analysis (Ntemou et al., 2023) of TMS studies on verb and sentence processing has nicely pointed to some variables within verb and sentence processing, attending to certain aspects such as regular and irregular verbs, syntax complexity, or action semantics. This kind of work can guide the items to choose during surgery.

## Relevance and conclusion

Why is this important? Electrostimulation time during awake surgery is limited. Long lists of items are usually applied, and there may be a way to determine the list of items optimal to the stimulated zone. Given those broad pools of items, a negative site may mean that the area is not functional or that the (≥2) items applied to that site could have been better chosen. The proposed item refinement and selection might seem subtle; however, they probably explain why linguistic errors are proportionally lower during stimulation than expected (Collée et al., 2023). We believe the most pertinent items within a task are probably left unknown, and the exploration might sometimes be blind. On the other hand, concrete knowledge of the items used would also be necessary in multitasking (Duffau et al., 2022), where inter-systems are addressed. Hence, increasing the sensitivity of the items requires selecting them according to a fundamental and well-tested rationale to adjust the actual probability of error. In turn, we would avoid false negatives and re-assure functional limits for surgery.

## Author contributions

ES: Writing – original draft, Writing – review & editing. SD: Writing – review & editing. CS: Writing – review & editing.
